# Rituximab in glomerular diseases: a case series and narrative review

**DOI:** 10.1590/2175-8239-JBN-2021-0120

**Published:** 2021-12-03

**Authors:** Inês Duarte, João Oliveira, Cristina Outerelo, Iolanda Godinho, Marta Pereira, Paulo Fernandes, Sofia Jorge, Joana Gameiro

**Affiliations:** 1 Centro Hospitalar Universitário Lisboa Norte, Serviço de Nefrologia e Transplantação Renal, Lisboa, Portugal.

**Keywords:** Glomerulonephritis, Rituximab, Immunosuppression, Remission Induction, Recurrence, Glomerulonefrites, Rituximab, Imunossupressão, Indução de Remissão, Recidiva

## Abstract

**Introduction:**

The use of Rituximab (RTX) in glomerular diseases (GD) has increased in the past years, although it is still only used in a small fraction of patients.

**Methods:**

A single center retrospective study of adult patients with membranous nephropathy (MN), focal segmental glomerulosclerosis (FSGS), lupus nephritis (LN), and vasculitis treated with RTX as first or second-line therapy was conducted at our center from 2010 to 2020.

**Results:**

We identified 19 patients; 36.8% had MN and 25.0% each had FSGS, LN, and vasculitis. RTX was first-line therapy in 26.3% of patients and in 73.7% it was second-line therapy. Mean follow-up time was 7.7 ± 7.2 years. In MN, 2 patients (28.6%) had complete remission (CR), 2 patients (28.6%) had partial remission (PR), and 3 patients (42.9%) had no response (NR). In FSGS, 2 patients (50.0%) presented CR, 1 patient (25.0%) had no response, and 1 patient had renal deterioration. Two patients (50.0%) had a LN class IV with a CR after RTX, 1 patient with LN class IIIC/V had no response, and 1 patient with LN class II had renal deterioration. In vasculitis, 3 patients (75.0%) presented CR and 1 patient had PR. Infusion reactions were present in 2 patients (10.5%) and one patient had multiple infectious complications.

**Conclusions:**

The efficacy of RTX in treating different types of immune-mediated GD has been demonstrated with different response rates, but an overall safe profile. In our case series, the results are also encouraging. Longitudinal studies are needed to better understand the effect of RTX in GD.

## Resumo

**Introdução:**

O uso de Rituximab (RTX) em doenças glomerulares (DG) aumentou nos últimos anos, embora ainda utilizado apenas em uma pequena fração de pacientes.

**Métodos:**

Conduzimos em nosso centro, de 2010-2020, um estudo retrospectivo de único centro de pacientes adultos com nefropatia membranosa (NM), glomeruloesclerose segmentar focal (GESF), nefrite lúpica (NL) e vasculite tratada com RTX como terapia de primeira ou segunda linha.

**Resultados:**

Identificamos 19 pacientes; 36,8% tinham NM; 25,0% cada apresentava GESF, NL e vasculite. RTX foi terapia de primeira linha em 26,3% dos pacientes e em 73,7% foi terapia de segunda linha. O tempo médio de acompanhamento foi 7,7 ± 7,2 anos. Em NM, 2 pacientes (28,6%) tiveram remissão completa (RC), 2 pacientes (28,6%) remissão parcial (RP), e 3 pacientes (42,9%) não tiveram resposta (NR). Na GESF, 2 pacientes (50,0%) apresentaram RC, 1 paciente (25,0%) não teve resposta e, 1 paciente, deterioração renal. Dois pacientes (50,0%) apresentaram NL classe IV com RC após RTX, 1 paciente com NL classe IIIC/V não teve resposta, e 1 paciente com NL classe II apresentou deterioração renal. Na vasculite, 3 pacientes (75,0%) apresentaram RC e 1 paciente RP. Reações à infusão ocorreram em 2 pacientes (10,5%) e um paciente apresentou múltiplas complicações infecciosas.

**Conclusões:**

A eficácia do RTX em tratar diferentes tipos de DG imunomediada tem sido demonstrada com diferentes taxas de resposta, mas com perfil geral seguro. Em nossa série de casos, os resultados também são encorajadores. Estudos longitudinais são necessários para compreender melhor o efeito do RTX na DG.

## Introduction

Glomerular diseases (GD) are an important cause of chronic kidney disease in most countries and more frequently affect younger patients^1^. They are a heterogeneous group of diseases with different presentations, clinical courses and outcomes, and the current treatment remains insufficient, with several adverse effects^2^.

Recent insights into the underlying biology and pathogenesis of human GD combined with scientific advances have enabled the identification of novel targeted interventions. GD include both renal-limited diseases and nephritides that develop as part of a systemic disorder. The pathogenesis is generally attributed to autoimmunity, exacerbated by a local renal immune and inflammatory response. Recent research has focused on the roles of innate immunity and B cells in auto- and alloimmune responses in different GD and in renal transplantation. T cell autoreactivity is B cell-dependent through autoantigen presentation and co-stimulatory support^3^, and autoantigen-specific B cells and plasmoblasts have been identified at inflammation sites in GD^4^.

This highlights the multiple roles of B cells in immune dysregulation, inflammation, and autoantibody synthesis^5^ and has led to the administration of rituximab (RXT) in different types of GD. RXT also affects the kidney filtration barrier in recurrent FSGS by preserving sphingolipid-related enzymes that might affect actin cytoskeleton remodeling in podocytes^6^.

RTX is a chimeric antibody that binds specifically to the B-cell surface antigen CD20, a protein which is expressed on immature and mature B lymphocytes, but it is not found in early B-cell precursors or plasma cells^7^. RTX causes a rapid and sustained depletion of circulating and tissue-based B-cells^8^. B cell depletion induced by RTX is heterogeneous in the various autoimmune and lymphoproliferative disorders reflecting the different phenotypes and functional heterogeneity of human B cell populations that play specific pathogenic roles in inflammatory and neoplastic disorders^9^. It also appears to cross-react with sphingomyelin-phosphodiesterase-acid-like-3b (SMPDL-3b) affecting actin cytoskeleton remodeling in podocytes^6^. This may explain the variety of RTX responses in several GD.

We present a single-center's experience with the use of RTX for the treatment of GD regarding clinical presentation, immunosuppression protocol, outcomes, and complications, and also present a narrative review of the efficacy and safety of this therapy.

## Materials and Methods

The authors present a single center retrospective study of all patients with GD confirmed by biopsy treated with RTX at Centro Hospitalar Universitário Lisboa Norte (CHULN) between January 2010 and March 2020. Patients were followed until December 2020. CHULN is an academic and referral center located in Lisbon, Portugal. This study was approved by the Ethical Committee in agreement with institutional guidelines. Due to the retrospective and non-interventional nature of the study, informed consent was waived by the Ethical Committee.

Eligible patients were patients with GD confirmed by biopsy treated with RTX. The decision to administer RTX therapy was based upon clinical criteria and subjected to institutional drug subcommittee approval. Patients were informed about the preliminary data on the efficacy and potential side effects of RTX therapy in adult GD.

Clinical data were obtained through the review of patients' medical records, and included demographic data at diagnosis, before starting RTX therapy, and at last follow-up. Laboratory data included serum creatinine and urinary protein at diagnosis and on the last follow-up.

Nephrotic syndrome (NS) was defined as 24-h urine protein >3.5 g/day with serum albumin <3 g/dL, hyperlipidemia and edema. Patients with 24-h urinary protein >1.5 g/day with significant edema were considered to have sub-nephrotic proteinuria and were treated with immunosuppression according to the clinical experience of our center. Rapidly progressive renal failure (RPRF) was defined as a decrease in renal function that progressed rapidly within a few weeks or months to renal failure and was accompanied by urinary findings of nephritis. Complete remission (CR) was defined as 24-h urinary protein <500 mg/day and partial remission (PR) as 24-h urinary protein between 500 mg and 3.5 g/day with at least 50% reduction in proteinuria from the time of initiation of therapy with estimated glomerular filtration rate (eGFR) maintained within 25% of the baseline. Non-responders (NR) were defined by <50% decrease in proteinuria with or without sustained decline in eGFR. Renal deterioration was defined as sustained decline in eGFR ≥ 50% from baseline documented at least twice. Steroid-dependent (SD) patients were defined by the presence of at least one relapse during steroid tapering.

### Treatment protocol

RTX was administered as an intravenous (IV) infusion of 3-4 h. Premedication included 1 g oral paracetamol, 100 mg hydrocortisone IV, and 25 mg oral hydroxyzine. The prescribed dose of RTX was based on the attending physician discretion and varied across time points. Rituximab was administered as four weekly pulses of 375 mg/m^2^ or as two fixed doses of 1000 mg on days 1 and 15.

Patients were closely monitored for infusion-related reactions such as headache, chills, fever, rash, or hypotension. Co-trimoxazole prophylaxis was given to all patients for at least 6 months after RTX therapy. All patients received diuretics, statin, angiotensin-converting enzyme inhibitor, and/or angiotensin receptor antagonist, as deemed appropriate to control edema, blood pressure, proteinuria, and hyperlipidemia.

### Statistical methods

Categorical variables were described as the total number and percentage for each category and continuous variables were described as the mean ± standard deviation. Statistical analysis was performed with the statistical software SPSS for windows (version 21.0).

## Results

**Figure 1:**
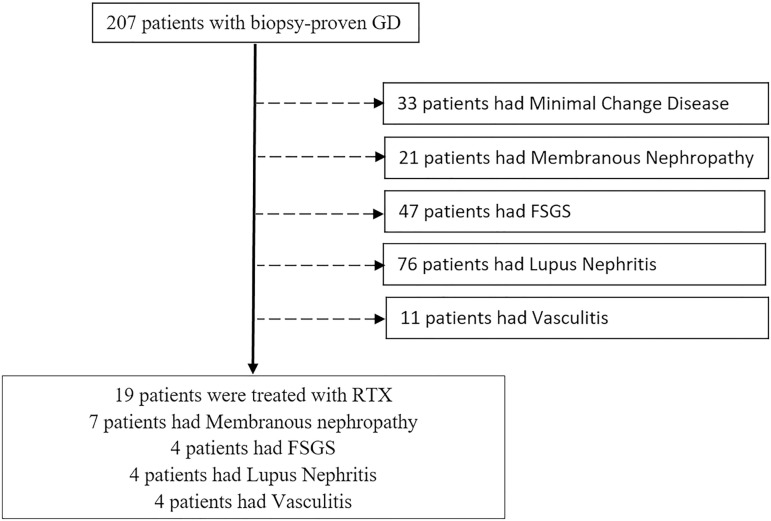
Flow chart of patient selection.

Overall, during the study period, 207 patients had GD confirmed by biopsy. As shown in Figure 1, 188 patients with biopsy-proven GD were excluded based on treatment with other immunosuppression regiments that did not include RTX. We studied a final cohort of 19 patients. The mean age of patients was 43.8 ± 21.2 years and the majority were male (n=11, 57.9%). Table 1 summarizes patient characteristics.

**Table 1:** Patients characteristics

Characteristic	Patients (n= 19)
Age at onset (years), mean ± SD	43.8 ± 21.2
Male gender, n (%)	11 (57.9)
*Clinical presentation, n* (%)	
Nephrotic syndrome	11 (57.9)
Subnephrotic proteinuria	4 (21.1)
RPRF	4 (21.1)
*Histologic diagnosis, n* (%)	
MN	7 (36.8)
FSGS	4 (21.1)
LN	4 (21.1)
Vasculitis	4 (21.1)
*Laboratory at presentation*	
Baseline serum creatinine (mg/dL), mean ± SD	1.5 ± 1.7
Baseline urinary protein (g/day), mean ± SD	4.9 ± 4.5
*Immunosuppressive first-line therapy, n* (%)	
Rituximab as first therapy, n (%)	5 (26.3)
Long-term prednisolone	15 (78.9)
Calcineurin inhibitors	6 (31.6)
Cyclophosphamide	7 (36.8)
Mycophenolate mofetil	5 (26.3)
Methotrexate	1 (5.3)
*Outcome after RTX*	
Complete remission, n (%)	9 (47.4)
Partial remission, n (%)	3 (15.8)
No response, n (%)	5 (26.3)
Renal deterioration, n (%)	2 (10.5)
Serum creatinine after rituximab (mg/dL), mean ± SD	1.6 ± 1.4
Urinary protein after rituximab (g/day), mean ± SD	1.9 ± 3.1
Follow-up, years	7.7 ± 7.2

*FSGS:* focal segmental glomerulosclerosis, *MN:* membranous nephropathy, *LN:* lupus nephritis, *RPRF:* rapidly progressive renal failure.

The clinical presentation at GD diagnosis was nephrotic syndrome in 11 patients (57.9%), subnephrotic proteinuria in 4 patients, and rapidly progressive renal failure in 4 patients (21.1%).

Seven patients had primary membranous nephropathy (MN) (36.8%), four patients had lupus nephritis (LN) (21.1%), four patients had focal segmental glomerulosclerosis (FSGS) (21.1%), and four patients had ANCA-vasculitis (21.1%).

At presentation, serum creatinine was 1.5 ± 1.7 mg/dL and the baseline 24-h urine protein was 4.9 ± 4.5 g.

Rituximab was first-line therapy in 5 patients (26.3%). The remaining patients received prior immunosuppressive therapy with corticosteroids (Pd) (n=15, 78.9%), calcineurin inhibitors (CNI) (n=6, 31.6%), cyclophosphamide (Cp) (n=7, 36.8%), mycophenolate mofetil (MMF) (n=5, 26.3%), and methotrexate (MTX) (n=1, 5.3%). Mean follow-up time was 7.7 ± 7.2 years. After RTX, a total of nine patients (47.4%) had complete remission, 3 patients (15.8%) had partial remission, 5 patients (26.3%) had no response, and 2 patients (10.5%) had renal deterioration during follow-up.

Baseline mean serum creatinine after RTX was 1.6 ± 1.4 mg/dL and the medium baseline 24-h urine protein was 1.9 ± 3.1 g.

### Primary membranous nephropathy

As shown in Table 2, 7 patients had primary membranous nephropathy (36.8%). The mean age of patients was 50.1 ± 23.1 years and the majority were male (n=5, 71.4%). The clinical presentation at diagnosis was NS in 6 patients (85.7%) and subnephrotic proteinuria in 1 patient (14.3%). At diagnosis, serum creatinine was 1.1 ± 0.4 mg/dL and the baseline 24-h urine protein was 6.5 ± 2.5g. One patient (14.3%) received RTX as first-line therapy. Six patients received prior IS therapy with long-term Pd (n=4, 57.1%), CNI (n=5, 71.4%), Cp (n=2, 28.6%), and MMF (n=1, 14.3%). Of these, 4 patients had complete remission and 2 patients had a partial remission. Apart from one patient, all presented one or more relapses prior to RTX (n=5/6, 83.3%). Mean follow-up time was 11.9 ± 9.5 years. After RTX therapy, a total of 2 patients (28.6%) had complete remission, 2 patients (28.6%) had partial remission and 3 patients (42.9%) had no response during follow-up. Baseline mean serum creatinine after RTX was 1.2 ± 0.5 mg/dL and the medium baseline 24-h urine protein was 2.5 ± 2.8 g.

**Table 2:** Treatment details of each individual patient

No	Age at onset (years)	Gender	GN	SCr at diagnosis (mg/dL)	Urinary protein at diagnosis (g/day)	Previous IS	Response before RTX	Number of relapses prior to rituximab	Follow-up after RTX therapy (years)	SCr follow-up (mg/dL)	Urinary protein follow-up (g/day)	Status at last follow-up	Complications of RTX therapy
1	24	M	MN	0.7	8.0	Pd, CyA, TAC, Cp	CR	2	31	0.9	1.1	PR	No
2	35	F	MN	0.8	3.5	Pd, CyA	CR	1	4	0.6	0.9	PR	No
3	76	M	MN	1.3	7.6	CyA, MMF	PR	1	13	1.9	5.4	NR	No
4	76	M	MN	1.3	9.3	Pd, CyA	CR	1	6	1.9	7.5	NR	No
5	28	F	MN	0.7	2.6	CyA	PR	0	8	0.8	2.4	NR	No
6	42	M	MN	1.5	6.6	Pd, Cp	CR	1	16	1.1	0.3	CR	No
7	70	M	MN	1.4	8.0	RTX	*	0	5	1.3	0.2	CR	infusion reactions
8	37	F	FSGS	1.7	0.7	Cp	NR	0	5	2.3	0.1	RD	No
9	7	M	FSGS	0.2	3.7	Pd, TAC	PR	0	13	0.8	7.0	NR	No
10	23	M	FSGS	0.8	11.8	Pd, MMF	CR	1	8	0.8	0.5	CR	No
11	15	M	FSGS	0.4	3.9	Pd	SD	0	4	0.6	0.1	CR	No
12	53	F	LN	0.9	1.2	Pd	SD	0	1	1.1	1.3	NR	No
13	26	F	LN	0.8	3.8	Pd, Cp, MMF	PR	0	13	0.8	0.0	CR	respiratory infection
14	47	F	LN	0.5	1.0	Pd, MTX, MMF	PR	1	7	0.6	0.0	CR	No
15	32	M	LN	2.4	16.9	Pd, Cp, MMF	PR	1	7	6.7	10.0	RD	No
16	66	M	V	8.0	0.0	Pd, Cp, RXT	*	0	2	3.3	0.1	PR	No
17	66	M	V	1.5	3.1	Pd, RTX	*	0	3	1.6	0.2	CR	infusion reactions
18	46	F	V	2.5	0.4	Pd, Cp, RXT	*	0	1	1.2	0.0	CR	No
19	63	F	V	1.9	0.1	Pd, RTX	*	0	0	1.4	0.1	CR	No

Cp: cyclophosphamide; *CyA*: cyclosporine A; *CR*: complete remission; *F*: Female; *FSGS*: focal segmental glomerulosclerosis; *MN*: membranous nephropathy; *LN*: lupus nephritis; *M*: male; *MTX*: metotrexato; *MMF*: mycophenolate mofetil; *NR*: no-response; *Pd*: prednisolone; *PR*: partial remission; *RPRF*: rapidly progressive renal failure; *RD*: renal deterioration; *RTX*: rituximab; *SD*: Steroid-dependency; *V*: vasculitis;

* RTX as first therapy.

### Focal segmental glomerulosclerosis

Four patients presented with FSGS (21.1%). The mean age of patients was 20.5 ± 12.8 years and the majority were male (n=3, 75.0%). The clinical presentation at diagnosis was NS in 3 patients (75.0%) and renal deterioration in 1 patient (25.0%). At diagnosis, serum creatinine was 0.8 ± 0.7 mg/dL and the baseline 24h- urine protein was 5.0 ± 4.7 g. All patients received prior IS therapy with long-term Pd (n=3, 75.0%), CNI (n=1, 25.0%), Cp (n=1, 25.0%), and MMF (n=1, 25.0%). One patient had complete remission with previous IS, 1 patient had partial remission, 1 patient had steroid dependency and 1 patient had no response. The patient with complete remission presented one relapse prior to RTX (25.0%). Mean follow-up time was 7.5 ± 4.0 years. After RTX therapy, two patients (50.0%) presented complete remission, one patient (25.0%) had no response, and one had renal deterioration during follow-up. Baseline mean serum creatinine after RTX was 1.1 ± 0.8 mg/dL and the mean baseline 24-h urine protein was 1.9 ± 3.4 g.

### Lupus nephritis

Four patients presented LN (21.1%). The mean age of patients were 39.5 ± 12.6 years and the majority were female patients (n=3, 75.0%). The clinical presentation at diagnosis was NS in 2 patients (50.0%) and subnephrotic proteinuria in 2 patients (50.0%). At diagnosis, mean serum creatinine was 1.2 ± 0.9 mg/dL and the baseline 24-h urine protein was 5.7 ± 7.6 g. All patients received previous IS with long-term Pd (n=4, 100.0%), Cp (n=2, 50.0%), MMF (n=3, 75.0%), and MTX (n=1, 25.0%). One patient was steroid-dependent and the other three were on partial remission. Two patients (50.0%) had a LN class IV with a complete remission after RTX, 1 patient had LN class IIIC/V on renal biopsy and had no response to RTX and 1 patient presented LN class II and had renal deterioration during follow-up. Mean follow-up time was 7.0 ± 4.9 years. Baseline mean serum creatinine after RTX was 2.3 ± 2.9 mg/dL and the mean baseline 24-h urine protein was 2.8 ± 4.8 g.

### ANCA-vasculitis

In the 4 patients with ANCA-vasculitis, RTX was administered as a first line therapy in combination with prednisolone and cyclophosphamide in 2 patients (50.0%). The mean age of patients was 60.3 ± 9.6 years and 50.0% of patients were male. The clinical presentation at diagnosis of all patients was rapidly progressive renal failure. At diagnosis, mean serum creatinine was 3.5 ± 3.0 mg/dL and the baseline 24-h urine protein was 0.9 ± 1.5 g. Three patients (75.0%) presented complete remission and 1 patient had partial remission. Mean follow-up time was 1.5 ± 1.3 years. Baseline mean serum creatinine after RTX was 1.9 ± 0.9 mg/dL and the mean baseline 24-h urine protein was 0.1 ± 0.1 g.

### Adverse effects

The majority of patients (n=17, 89.5%) tolerated RTX treatment without any report of adverse effects. Two patients (10.5%) experienced infusion reactions (skin rash, throat irritation, chest tightness, difficulty breathing, hypotension, bradycardia, and body aches), which were self-limiting events and resolved with infusion rate reduction. One patient presented 3 episodes of severe respiratory infections after RTX that responded to a course of oral antibiotics. This patient was previously immunosuppressed with cyclophosphamide.

## Discussion

Our results support the safety and efficacy of RTX for inducing remission of different types of immune-mediated glomerular diseases. There were very few immediate side effects due to infusion of RTX and the follow-up during the study was uneventful, particularly in patients who received RTX as a first-line therapy.

This anti-CD20 monoclonal antibody has an immunosuppressive role by depleting B cells, decreasing antibodies and cytokines production, and altering the process of antigen presentation. Furthermore, recent research has demonstrated that the podocyte cytoskeleton may be a direct target for RTX, through modulation of IL-17 production and actin cytoskeleton stabilization by the connection with sphingomyelin phosphodiesterase acid-like 3b, leading to podocyte apoptosis^10^. RTX causes a rapid and sustained depletion of circulating and tissue-based B-cells^8^. In most patients, this dose-dependent effect persists for 2-3 months; in some, it is maintained for 6 months, followed by a slow recovery with median B-cell levels returning to normal by 12 months^8^. The recognition of the multiple roles of B cells in immune dysregulation, inflammation, and autoantibody synthesis^5^ has led to the administration of RXT in different types of GD.

A final cohort of nineteen patients with GD confirmed on biopsy were treated with RTX of which twelve (63.2%) had a complete or partial remission suggesting the therapeutic role of RTX in several types of GD.

RTX was mostly used in patients with MN. MN is one of the main causes of NS in adults^11^, and in approximately 80% of the cases its pathophysiology is related to the presence of autoantibodies against M-type phospholipase A2 receptor 1 (PLA2R1) and thrombopondin type-1 domain-containing 7A (THSD7A), providing evidence of the role of B cell activation in MN^12^. Indeed, the initial experience with RTX in MN as a second-line immunosuppressive therapy was positive^13^. GEMRITUX^14^ was the first trial that compared two doses of RTX (375 m/m^2^) with no immunosuppressive therapy after an adequate period of conservative management. Although there was no significant difference between the two groups at 6 months, the remission rate at the extended follow-up was significantly higher in the RTX group (64.9% versus 34.2%, P < 0.01), with no significant adverse effects compared with untreated patients.

More recently, the MENTOR trial^15^ demonstrated complete or partial remission after 24 months in 60% patients of the RTX cohort as a first-line therapy compared to 20% partial remission in CyA cohort, without any complete remission. In our cohort, we experienced complete or partial remission in 57.1% (n=4/7) of cases, which was stable during follow-up. The majority of our patients had received prior immunosuppressive therapy and one patient received RTX as first-line therapy. Our response rate was similar to other studies, which have shown remission in 60-70% of patients^16^^,^ 17.

RTX was also reported to be effective in inducing remission in adult and pediatric patients with multi-relapsing NS secondary to minimal change disease (MCD), and also in those with steroid-dependent NS^18^. Furthermore, RTX could induce remission in patients with recurrent FSGS after transplantation and in FSGS in native kidneys^19^. Gulati et al.^20^ reported the efficacy of RTX therapy for childhood steroid-dependent or steroid-resistant NS with a response rate of 83 and 49%, respectively. In a report by Ruggenenti et al^21^. of 10 children and 20 adults with steroid-dependent and frequently relapsing NS, RTX was effective in preventing recurrences and reduced the need for immunosuppression, and halted disease-associated growth deficit in children. The overall remission rate of RTX therapy was 53.6%, with complete remission in 42.9% in a recent meta-analysis by Hansrivijit et al^22^. of five studies with FSGS patients (n = 51). They also concluded that RTX may be considered an additional treatment to the standard therapy for adult patients with FSGS and MCD.

In this cohort, 50% of patients with FSGS had a steady and complete remission with rituximab therapy. All patients had received prior immunosuppressive therapy and in one case the primary clinical presentation was progressive renal failure, which may have contributed to the absence of response to RTX.

Therapeutic benefit of RTX has been reported in LN patients where conventional treatment had failed^23^. Condon et al^24^. reported a complete and partial remission of 52 and 34%, respectively, in a prospective cohort of LN patients treated with 2 doses of rituximab (1 g) and methyl prednisolone (500 mg) on days 1 and 15. In a pediatric cohort, flare-free survival was significantly higher at 36 months with RTX compared with MMF and CYC (100% for RTX vs. 83% for MMF and 53% for CYC, p = 0.006)^25^. Zhong et al^26^. published a meta-analysis of five randomized controlled trials that analyzed clinical remission with RTX therapy and concluded that treatment with RTX was associated with a higher total remission, defined by the combination of CR plus PR.

In our cohort, RTX was administered in refractory LN class IV patients and we had a 50% rate of complete remission in both patients.

Although B cells are rarely found in the kidney in ANCA vasculitis (AAV), the positive effect of RTX suggests that the therapeutic effect is either through reduction in ANCA levels or through indirect effects on autoreactive T cells. The latter is probably more likely because RTX is effective even in ANCA-negative vasculitis, and effects of RTX on B cell:T cell co-stimulation has also been shown in rheumatoid arthritis and systemic lupus erythematosus^28^.

Several prospective and retrospective studies have reported a high level of efficacy of RTX in relapsing or refractory vasculitis^29^^,^ 30. Two randomized controlled trials have evaluated RTX in induction treatment of AAV. In the RAVE trial^31^, RTX was compared with oral Cp and induced a complete remission of disease at 6 months in 64% versus 53% in patients treated with Cp. In the RITUXVAS trial^30^, RTX was compared with IV Cp and demonstrated a sustained complete remission in 76% compared with 82% in the Cp arm. Both trials demonstrated similar response rates following induction treatment with RTX, supporting the use on RTX as a first-line therapy.

In our cohort, RTX were administered as first-line therapy in all patients with vasculitis and 75% of these achieved a complete remission, which is in line with the results found in the literature.

Our study was conducted in a single center and was retrospective, which are important limitations. Indeed, the small number of patients in each category of GD limits the generalizability of our results. Second, we did not routinely perform CD20 cell count, which would have been important to understand response rates after RTX in different GD. Third, differences in treatment protocols between the main studies and our case series require caution interpretation of these results.

Nevertheless, our study provided significant insight on the use of RTX in different GD and suggests a differential response to rituximab therapy, which may provide the background for future pathophysiology studies.

## Conclusion

The use of RTX in GD has increased in the past years, although it is still only used in a small proportion of patients. The efficacy in treating different types of immune-mediated GD with RTX has been demonstrated with different response rates, but with an overall safe profile. In our case series, the results are also encouraging. Longitudinal studies are needed to better understand the effect of RTX in GD.
